# Novel Insights into MEG3/miR664a-3p/ADH4 Axis and Its Possible Role in Hepatocellular Carcinoma from an in Silico Perspective

**DOI:** 10.3390/genes13122254

**Published:** 2022-11-30

**Authors:** Shreyas H. Karunakara, Lakshana D. Puttahanumantharayappa, Nirmala G. Sannappa Gowda, Varsha D. Shiragannavar, Prasanna K. Santhekadur

**Affiliations:** Department of Biochemistry, Center of Excellence in Molecular Biology & Regenerative Medicine, JSS Medical College, JSS Academy of Higher Education and Research, Mysore 570015, India

**Keywords:** MEG3, miR-664a-3p, alcohol dehydrogenase 4, tumor suppressor, hepatocellular carcinoma, mean binding energy

## Abstract

Hepatocellular carcinoma (HCC) is a complex disease involving altered interactomes of transcripts and proteins. MicroRNAs (miRNAs) are small-noncoding RNAs that can interact with specific gene transcripts and an array of other vital endogenous non-coding RNAs (lncRNAs) that can influence gene expression. Maternally Expressed Gene 3 (MEG3) is an imprinted lncRNA that is reported to be downregulated in HCC (in both cell lines and tumors). Alcohol Dehydrogenase 4 (ADH4) is a well-known prognostic protein biomarker for predicting the survival outcomes of patients with hepatocellular carcinoma whose expression is regulated by miR-664a-3p, which is upregulated in HCC. In this study, we performed a battery of robust and systematic in silico analyses to predicate the possible lncRNA–miRNA interactions between MEG3, miR-664a-3p, and ADH4. miRNA–mRNA and lncRNA–miRNA hybrid structures were primarily obtained, and the minimum free energies (MFEs) for the 3′UTR (Untranslated Regions) of ADH4-miR-664a-3p and the 3′UTR of MEG3-miR-664a-3p interactions were assessed to predict the stability of the obtained RNA heteroduplex hybrids. The hybrid with the least minimum free energy (MFE) was considered to be the most favorable. The MFEs were around −28.1 kcal/mol and −31.3 kCal/mol for the ADH4-miR-664a-3p and MEG3-miR-66a-3p RNA hybrids, respectively. This demonstrated that lncRNA-MEG3 might be a competitive endogenous RNA that acts as a molecular sponge for miR-664a-3p. In summary, our interaction analyses results predict the significance of the MEG3/miR-664a-3p/ADH4 axis, where MEG3 downregulation results in miR-664a-3p overexpression and the subsequential underexpression of ADH4 in HCC, as a novel axis of interest that demands further validation.

## 1. Introduction

Hepatocellular carcinoma (HCC) refers to the primary carcinoma of the liver and ranks as the sixth most common cancer affecting the human population [1]. According to the Global Cancer Observatory (https://gco.iarc.fr/ (accessed on 5 October 2022)), the national mortality burden in India for HCC stands at 2.4%, accounting for about 33,793 deaths. Preliminary causes include chronic liver conditions, such as Hepatitis B Virus (HBV) [2,3,4,5] and Hepatitis C Virus (HCV) [6,7] infections, cirrhosis, non-alcoholic fatty liver disease (NAFLD) [8,9,10,11], and non-alcoholic steatohepatitis (NASH) [11,12,13]. Additionally, several other epigenetic mechanisms involving long non-coding RNAs (lncRNAs), microRNAs (miRNAs), histone modifications, and DNA methylation are gaining importance in regulating various target genes vital to the development and progression of HCC [14].

Maternally Expressed Gene 3 (MEG3) is located on Chromosome 14q32.3. In humans, it is located on the imprinted *DLK1-MEG3* locus. The mouse ortholog commonly referred to as *Meg3* or Gene trap locus 2 (*Glt2)* is positioned on Ch 12 [15,16]. MEG3 encodes for an lncRNA, which is commonly expressed in normal tissues [17,18,19]. However, MEG3 expression is lost in cancers of the brain [20], breast [21], lung [21], cervix [21], and colon [22]. In HCC, MEG3 is underexpressed when compared to in normal liver tissues [23]. MEG3 underexpression has been validated in various HCC cell line models, such as PLC/PRF/5 [23], HepG2 [24], and Huh7 [25]. Overall, these experimental pieces of evidence suggest that MEG3 is downregulated and acts as a bona fide tumor suppressor in HCC.

MicroRNAs (miRNAs) play a crucial role in regulating the gene expression of target genes by binding to the 3′UTRs of target transcripts and degrading mRNA through RISC-mediated endonuclease activity [26,27]. Several studies have reported miR-664a-3p expression to be overexpressed in HCC [28]. A study by Wang in 2019 reported miR-664 to be overexpressed in HCC cell lines, as well as in HCC patients, adding to the progression and spread of HCC [29].

The alcohol dehydrogenase (ADH) superfamily spans five classes of dehydrogenases: class I (ADHIA, ADHIB, and ADHIC), class II (ADH4), class III (ADH5), class IV (ADH6), and class V (ADH7) [30]. ADH4 is primarily involved in the metabolism of aliphatic alcohols, including retinol, ethanol, and hydroxysteroid, and it occupies center stage in the hepatic regulation of the metabolism of various lipid peroxidation processes [31]. Previous studies have shown that ADH4 is significantly downregulated, and they have predicted its suitability as a prognostic marker in HCC [32,33,34].

In the present study, we employed a range of robust algorithms and bioinformatics tools to identify a novel axis that might be central to HCC development and progression. Based on RNA–RNA interactions, we predicted that MEG3 interacts with miR-664a-3p, resulting in the overexpression of ADH4 in normal liver, while the reverse effect occurs due to the underexpression of MEG3 in HCC.

## 2. Materials and Methods

### 2.1. Retrieval of lncRNA, miR-664a, and ADH4 Sequences

Reference nucleotide sequences were obtained from standard databases. The gene coordinates for human lncRNA MEG3 were found to be Chr14: 100,826,108–100,861,026. The full-length nucleotide sequence of MEG3 (NC_000014.9: https://www.ncbi.nlm.nih.gov/nuccore/NC_000014.9?rport=fasta&from=100826108&to=100861026 (accessed 5 October 2022)) and the transcript of ADH4 (M15943.1: https://www.ncbi.nlm.nih.gov/nuccore/M15943.1?report=fasta (accessed 5 October 2022)) were obtained from the National Center for Biotechnology Information Nucleotide (Internet). Bethesda (MD): National Library of Medicine (US), National Center for Biotechnology Information; (1988)—Accession No. NC_000014.9, Homo sapiens Maternally Expressed Gene 3; (cited 5 October 2022). The mature sequence of miR-664a-3p (UAUUCAUUUAUCCCCAGCCUACA) was obtained from miRbase (https://www.mirbase.org/ (accessed 5 October 2022)) [35].

### 2.2. Expression of ADH4 in HCC

The next-generation sequencing (RNA-Seq) gene expression quantification data derived from the complete transcriptome profiling of the genes expressed in normal liver tissues (*n* = 70) and in HCC (*n* = 352) were downloaded from the TCGA-LIHC dataset from The Genomic Data Commons (GDC: https://gdc.cancer.gov/about-gdc) (data accessed on 5 October 2022) of the National Cancer Institute. The expression values of ADH4 in terms of transcripts per million from the whole transcriptome profile were obtained for which a differential expression analysis was performed on normal and HCC datasets (Supplementary Data 1: TCGA miR664a and ADH4 values.xlsx). The differential expression values were used to obtain box plots using Microsoft Excel. Further, the protein expression of ADH4 in the normal, as well as the TCGA-LIHC dataset, was obtained and analyzed using the Human Protein Atlas (HPA: https://www.proteinatlas.org/ (accessed on 5 October 2022)) [36]. The association of the differential expression of ADH4 in the normal and HCC datasets was determined in terms of overall survival (OS) and disease-free survival (DFS) using Gene Expression Profiling Interactive Analysis 2 (GEPIA 2.0: http://gepia2.cancer-pku.cn/ (accessed on 5 October 2022)) [37]. KM survival plots were plotted such that the true median expression values occurred with a confidence interval of 95%.

### 2.3. Differential Expression of miR-664a-3p and MEG3 in HCC

The miR-Seq quantification data were downloaded from the TCGA-LIHC dataset from the Genomic Data Commons portal (accessed on 5 October 2022). The expression values of miR-664a-3p in terms of transcripts per million in normal liver and HCC datasets were obtained from the whole transcriptome profile (Supplementary Data 2: TCGA miR664a and ADH4 values.xlsx). A differential expression analysis of miR-664a-3p in normal liver and HCC datasets were performed for which box plots were plotted. Further, mRNA expression was analyzed using The **U**niversity of **AL**abama at Birmingham **CAN**cer data analysis portal tool (UALCAN: http://ualcan.path.uab.edu/index.html (accessed on 5 October 2022)) [38]. For analyzing MEG3 expression in HCC, the TCGA-LIHC datasets were analyzed using UALCAN. The datasets for the differential expression of miR-664a-3p and lncRNA-MEG3 for normal and primary HCC were obtained from TCGA and analyzed using the UALCAN tool. The differential expression between the normal and tumor datasets was considered statistically significant if the means of differential expression for miRNA and lncRNA was *p* < 0.05 (95% CI).

### 2.4. Construction of miRNA–mRNA RNA Heteroduplex Hybrids

The interaction of miR-664a-3p and the 3′UTR of ADH4 mRNA was constructed using the BiBiserve2-RNAhybrid tool (https://bibiserv.cebitec.uni-bielefeld.de/rnahybrid/ (accessed on 5 October 2022)) [39]. The RNA heteroduplex hybrids were scored in terms of mean free energy (MFE). The top five RNA–RNA hybrids were considered for further analysis, and the hybrid with the least MFE was considered the best possible hybrid predicted.

### 2.5. Construction of lncRNA–miRNA RNA Heteroduplex Hybrids

The interaction of lncRNA MEG3 and miR-664a-3p was constructed based on sequence complementarity using the BiBiserve2-RNAhybrid tool. The top five hybrids were considered for further analysis, and the hybrid with the least MFE was considered the best possible hybrid predicted based on sequence complementarity.

### 2.6. Predicting the Accessibility of Binding Regions of MEG3-miRNA RNA Heteroduplex

Once the complementary sequences involved in the hybrid were identified, the actual binding of the lncRNA and miRNA was computed considering intramolecular interactions to provide a biophysically realistic model with accurate MFE using Freiburg RNA Tools: IntaRNA (http://rna.informatik.uni-freiburg.de/IntaRNA/Input.jsp (accessed on 5 October 2022)) [40,41].

## 3. Results

### 3.1. ADH4 Is Downregulated in HCC

**Figure 1:**
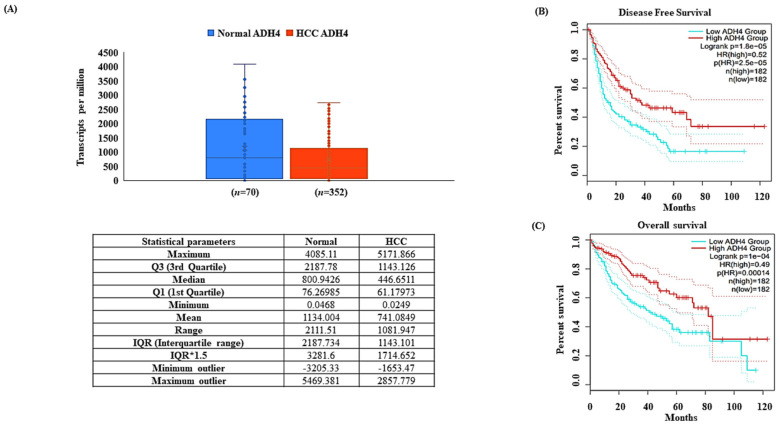
ADH4 is downregulated in hepatocellular carcinoma. ADH4 expression profiles (**A**). ADH4 mRNA expression box plot (statistical parameters included) (**B**). Overall survival of ADH4 in TCGA-LIHC datasets (**C**). Disease-free survival of ADH4 in TCGA-LIHC datasets.

The differential expression analysis of the expression values derived from the GDC portal for ADH4 in TCGA-LIHC (Supplementary Data 1) pointed towards a significant downregulation in HCC (Figure 1A). Moreover, the immunohistochemistry (IHC) details of ADH4 from the HPA analysis of normal and HCC tissue samples clearly showed the protein to be downregulated in HCC as compared to its higher expression in normal liver tissues (Supplementary Figure S1: HPA.TIF). It was observed that, when compared to the normal datasets, the HCC tissue datasets showed lower or no expression for ADH4. The individual patient details and IHC characteristics can be seen in Table 1. The significance of ADH4 expression in HCC was scored in terms of OS and DFS. It was observed that the differential expression of ADH4 is significant both in terms of OS (Figure 1B) and DFS (Figure 1C). The KM plots for OS showed that higher ADH4 expression corresponded to better survival as compared to decreased ADH4 expression. This clearly shows that ADH4 is downregulated in terms of differential expression in HCC.

### 3.2. LncRNA-MEG3 Is Downregulated and miR-664a-3p Is Upregulated in HCC

**Figure 2:**
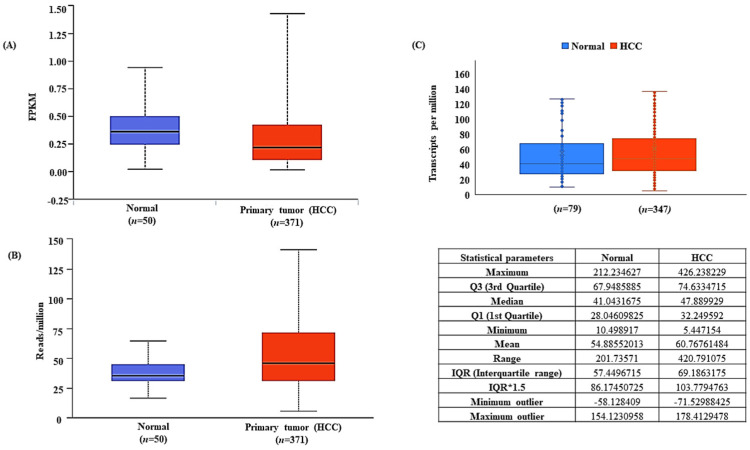
Differential expression of MEG3 and miR-664a-3p in TCGA-LIHC. (**A**) MEG3 is downregulated in HCC as compared to normal liver tissues in TCGA-LIHC. (**B**) miR-664a-3p is upregulated in HCC as compared to normal liver tissues in TCGA-LIHC. (**C**) Validation of transcriptome profile of miR-664a-3p in HCC (statistical parameters included).

The differential expression of lncRNA-MEG3 was obtained from TCGA-LIHC. The UALCAN analysis showed that MEG3 was downregulated in HCC tissues (*n* = 371) as compared to normal liver tissues (*n* = 50), with a significance of *p* = 7.0333E-06 (Figure 2A). Likewise, the UALCAN analysis of the differential expression of miR-664a-3p showed a significant upregulation of miRNA in HCC (*p* = 1.2608 × 10^−11^ (Figure 2B) (a value of *p* < 0.05 was considered statistically significant). Further, the expression values derived from the GDC Data Portal (Supplementary Data 2: TCGA miR664a and ADH4 values.xlsx) validated the upregulation of miR-664a-3p expression in HCC (Figure 2C).

### 3.3. ADH4 Is Targeted by miR-664a-3p in HCC

**Figure 3:**
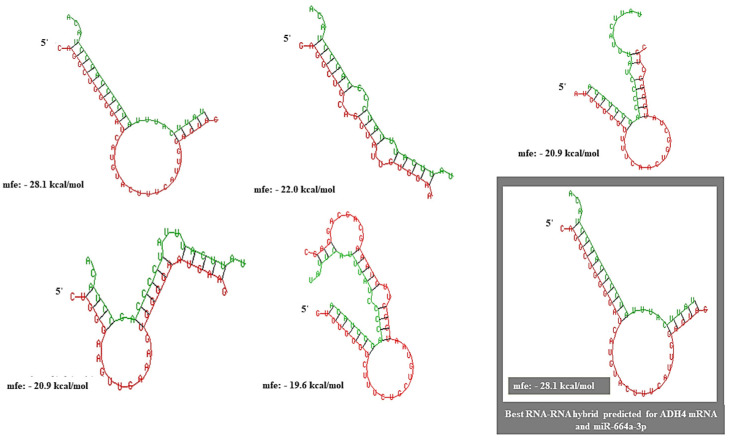
RNA heteroduplexes constructed for ADH4 mRNA and miR-664a-3p with the top five predicted miRNA–mRNA interactions. The best possible interaction had an MFE of −28.1 kcal/mol.

The probability of RNA–RNA interactions between miR-664a-3p and the 3′UTR of ADH4 was identified and scored using the RNA Interactome Database (RNAInter: http://www.rnainter.org/ (accessed on 5 October 2022)) on a scale of 0.0–1.0. The interaction had a score of 0.4571. The miRNA–mRNA interaction predicted was supplemented by strong published experimental evidence (PUBMED ID: 28374914). miRNA–mRNA heteroduplex hybrid structures were constructed using RNAhybrid, and the best-predicted structure had an MFE of −28.1 kcal/mol (Figure 3). 

### 3.4. MEG3 Acts as a Competitive Endogenous Sponge for miR-664a-3p

**Figure 4:**
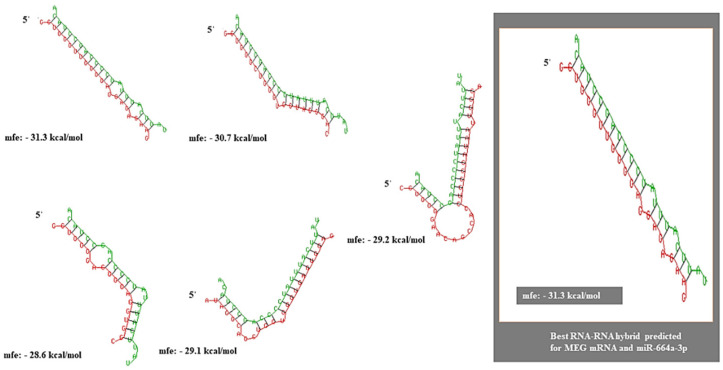
RNA heteroduplexes constructed for lncRNA-MEG3 and miR-664a-3p with the top five predicted lncRNA–miRNA interactions. The best possible model with an MFE of −31.3 kcal/mol is highlighted.

**Figure 5:**
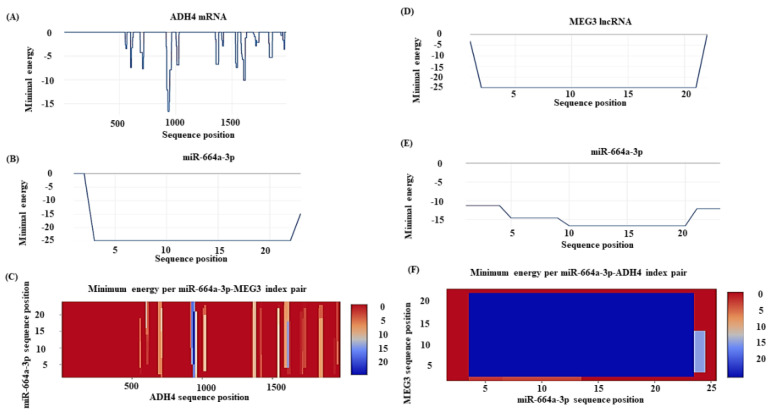
Prediction of accessible regions in RNA heteroduplexes. (**A**) Graph showing minimum free energy prediction for stable ADH4 mRNA. (**B**) Graph showing minimum free energy prediction for stable miR-664a-3p. (**C**) Minimum free energy prediction for the miRNA–mRNA hybrid. (**D**) Graph showing minimum free energy prediction for stable lncRNA MEG3 structure. (**E**) Graph showing minimum free energy graph for the stable miR-664a-3p structure. (**F**) Minimum free energy prediction for the lncRNA–miRNA hybrid.

The RNAInter analysis of lncRNA–miRNA predicted the interaction between MEG3-miR-664a-3p with a score of 0.528 on a confidence scale of 0.0–1.0. The miRNA–mRNA interaction predicted was supplemented by strong published experimental evidence (PUBMED ID: 28374914). Further, the RNA heteroduplex hybrid structures constructed using RNAhybrid predicted the best hybrid to have an MFE of −31.3 kcal/mol (Figure 4). IntaRNA predicted realistic model for the mRNA–miRNA interaction between the 10th–20th base positions of miR-664a-3p and the 931st–941st base positions of ADH4, with an overall MFE of −16.64 kcal/mol (Figure 5A–C). Moreover, a more realistic hybrid model based on the accessibility of nucleotides for base pairing predicted binding between the 3rd–22nd bases of miR-664a-3p and the 2nd–21st bases of MEG3, with an overall MFE of −24.75 kcal/mol (Figure 5D–F). The individual energy values for the interaction can be obtained from Supplementary Tables S1–S3: List of Supplementary Tables MEG3.pdf.

## 4. Discussion

RNA–RNA interactions involving lncRNAs and miRNAs occupy center stage in the post-transcriptional regulation of gene expression [42]. Various studies have revealed the significance of the tumor-suppressive effect of lncRNA MEG3 in HCC [42,43,44,45,46,47,48,49]. In our study, the datasets obtained from TCGA-LIHC showed a unanimous underexpression of MEG3 in HCC as compared to in normal liver, pointing out that MEG3 acts as a bona fide tumor suppressor in HCC.

miR-644 overexpression is also found to be associated with the downregulation of methionine adenosyl transferase and global hypomethylation in HCC [28], pointing towards the intricate effect and regulation of other epigenetic processes, such as DNA methylation by miRNAs. miR-664a-3p is reported to be upregulated in HepG2, Huh7, and MHCC97 HCC cell lines and is predicted to be associated with poor overall survival in HCC [29]. The analysis of the TCGA-LIHC dataset pointed towards a similar upregulation of miR-664a-3p in HCC.

ADH4 is a well-known prognostic biomarker for HCC [32]. Various studies have provided evidence that the downregulation of ADH4 is linked to the poor overall survival of HCC [50,51]. We mined the HPA-IHC data of ADH4, and they clearly show that ADH4 is underexpressed in HCC tissues. The Kaplan–Meier survival analysis inferred overall survival to be higher under the positive expression of ADH4, while the survival outcomes dropped under decreased ADH4 expression. Further, studies involving the validation of ADH4 in HCC using various methods, such as Western blot [32], RTqPCR [52], and DNA microarrays [53], have accurately predicted ADH4 to be downregulated. Additionally, ADH4 was predicted to be a prominent prognostic biomarker for the evaluation of immunotherapy efficiency in HCC patients [52].

Through our in silico analysis, we showed ADH4 to be a direct target for miR-664a-3p, wherein the structures of the miRNA–mRNA interaction between miR-664a-3p and the 3′UTR of ADH4 were identified and scored in terms of sequence complementarity and MFE. Likewise, similar models of interactions between miR-664a-3p and lncRNA-MEG3 were constructed based on sequence complementarity and MFE. We found that the mRNA–miRNA heteroduplex hybrid yielded a higher MFE (−28.1 kcal/mol) than a stable heteroduplex between miRNA and lncRNA, which yielded a lower MFE (−31.3 kCal/mol). Further, a more realistic model for mRNA–miRNA and lncRNA–miRNA interactions was predicted based on the accessibility of binding regions, which assigned an MFE of −16.64 kcal/mol for the miR-664a-3p-ADH4 interaction and an MFE of −24.75 kcal/mol for the MEG3-miR-664a-3p interaction. Based on the overall MFE predicted by three separate parameters (sequence complementarity, accessibility of binding regions, and MFE), it is inferred that MEG3 acts as a competitive sponge for miR-664a-3p.

**Figure 6:**
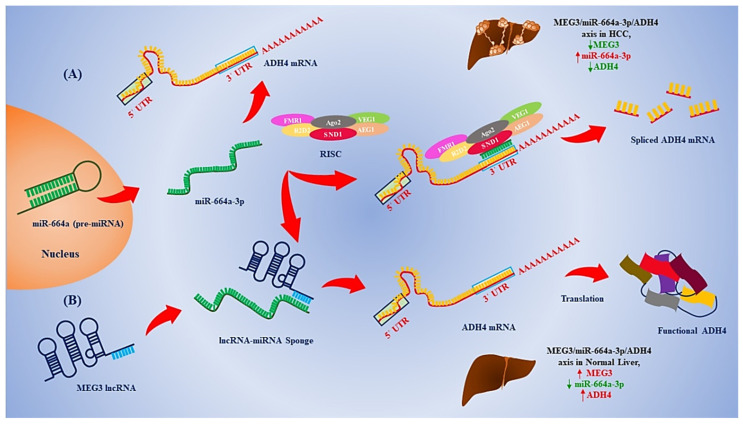
The overall mechanism of MEG3/miR-664a-3p/ADH4 axis. (**A**) In normal liver, MEG3 acts as a competitive molecular sponge for miR-664a-3p and prevents miR-664a-3p-mediated RISC targeting and degradation of ADH4 mRNA. (**B**) In HCC, MEG3 is downregulated resulting in miR-664a-3p expression and binding to ADH4 mRNA, causing subsequent downregulation of ADH4 mRNA through RISC mediated cleavage of the mRNA.

Based on the previous discussion, it can be concluded that the MEG3/miR-664a-3p/ADH4 axis is vital in the process of HCC carcinogenesis. In normal liver, MEG3 is not repressed and can act as a competitive sponge for miR-664a-3p, and MEG3 also acts as a molecular checker of miRNA expression and, in turn, prevents its binding to the 3′UTR of ADH4 mRNA (Figure 6A). However, in HCC, due to MEG3 downregulation, miR-664a-3p is upregulated and is free to bind to the 3′UTR of ADH4 mRNA, resulting in RNA-induced silencing of ADH4 (Figure 6B). Interestingly, we did find a study validating the existence of such an axis in HCC [54]. However, our study predicts miRNA–mRNA and miRNA–lncRNA hybrids based on MFE, which is novel and can serve as a template for in silico predictions of other RNA–RNA interactions that can form the basis for further in vitro validations in HCC.

## Figures and Tables

**Table 1:** Patient characteristics for HPA ADH4 expression in HCC and normal liver tissues.

Patient ID	Sex	Age	State	Staining	Intensity	Quantity	More Details
**3402**	Female	54	Normal	Medium	Moderate	>75%	https://www.proteinatlas.org/ENSG00000198099-ADH4/tissue/liver#img (accessed on 5 October 2022)
**3222**	Female	63	Normal	Medium	Moderate	>75%	
**2429**	Male	55	Normal	Medium	Moderate	>75%	
**3215**	Female	61	HCC	Low	Weak	25–75%	https://www.proteinatlas.org/ENSG00000198099-ADH4/pathology/liver+cancer#img (accessed on 5 October 2022)
**2766**	Female	73	HCC	Not detected	Negative	None	
**2177**	Female	58	HCC	Low	Weak	25–75%	
**3196**	Male	65	HCC	Not detected	Negative	None	
**2280**	Male	80	HCC	Medium	Moderate	25–75%	
**2556**	Male	72	HCC	Low	Weak	25–75%	
**3346**	Female	73	HCC	Medium	Moderate	25–75%	
**3477**	Male	67	HCC	Low	Weak	25–75%	

## Data Availability

Data available in a publicly accessible repository that does not issue DOIs. Publicly available datasets were analyzed in this study. This data can be found here: [https://portal.gdc.cancer.gov/] (accessed on 5 October 2022).

## Supplementary Materials

The following supporting information can be downloaded at: https://www.mdpi.com/article/10.3390/genes13122254/s1, Figure S1: HPA-Immunohistochemistry data for ADH4 expression in HCC; Supplementary Data 1: TCGA miR-664a expression in terms of transcripts per million; Supplementary Data 2: TCGA ADH4 expression values in terms of Transcripts per million Supplementary Table S1: Energies of individual nucleotides of MEG3 involved in miRNA-lncRNA interaction; Supplementary Table S2: Energies of individual nucleotides of miR-664a-3p involved in miRNA-lncRNA interaction; Supplementary Table S3: Final MFEs of nucleotides involved in miR-664a-3p-MEG3 interaction.
