# Supplementary material for: Novel Insights into MEG3/miR664a-3p/ADH4 Axis and Its Possible Role in Hepatocellular Carcinoma from an in Silico Perspective
Source: Genes (Basel). 2022 Nov 30;13(12):2254. doi: 10.3390/genes13122254 (PMC9778073; doi:10.3390/genes13122254)
Supplement: Supplementary file 1 [file genes-13-02254-s001.zip › Supplementary Tables S1¿CS3.pdf]

Supplementary tables

Supplementary Table S1. Energies of individual nucleotides of MEG3 involved in miRNA-lncRNA interaction.

Nucleotide Position	ENERGY
1	-3.06
2	-24.75
3	-24.75
4	-24.75
5	-24.75
6	-24.75
7	-24.75
8	-24.75
9	-24.75
10	-24.75
11	-24.75
12	-24.75
13	-24.75
14	-24.75
15	-24.75
16	-24.75
17	-24.75
18	-24.75
19	-24.75
20	-24.75
21	-24.75
22	0

Supplementary Table S2. Energies of individual nucleotides of miR-664a-3p involved in miRNA-lncRNA interaction.

**Nucleotide Position**	**ENERGY**
1	0
2	0
3	-24.75
4	-24.75
5	-24.75
6	-24.75
7	-24.75
8	-24.75
9	-24.75
10	-24.75
11	-24.75
12	-24.75
13	-24.75
14	-24.75
15	-24.75
16	-24.75
17	-24.75
18	-24.75
19	-24.75
20	-24.75
21	-24.75

Supplementary Table S3. Final MFEs of nucleotides involved in miR-664a-3p-MEG3 interaction.

		0	0	0	0	0	0	0	0	0	0	0	0	0	0	0	0	0	0	0	0	0	0	0	
1		0	0	-2.36	-2.36	-2.36	-3.06	-3.06	-3.06	-3.06	-3.06	-3.06	-3.06	0	0	0	0	0	0	0	0	0	0	0	0	1
2		0	0	-24.75	-24.75	-24.75	-24.75	-24.75	-24.75	-24.75	-24.75	-24.75	-24.75	-24.75	-24.75	-24.75	-24.75	-24.75	-24.75	-24.75	-24.75	-24.75	-24.75	0	0	2
3		0	0	-24.75	-24.75	-24.75	-24.75	-24.75	-24.75	-24.75	-24.75	-24.75	-24.75	-24.75	-24.75	-24.75	-24.75	-24.75	-24.75	-24.75	-24.75	-24.75	-24.75	-14.74	0	3
4		0	0	-24.75	-24.75	-24.75	-24.75	-24.75	-24.75	-24.75	-24.75	-24.75	-24.75	-24.75	-24.75	-24.75	-24.75	-24.75	-24.75	-24.75	-24.75	-24.75	-24.75	-14.74	0	4
5		0	0	-24.75	-24.75	-24.75	-24.75	-24.75	-24.75	-24.75	-24.75	-24.75	-24.75	-24.75	-24.75	-24.75	-24.75	-24.75	-24.75	-24.75	-24.75	-24.75	-24.75	-14.74	0	5
6		0	0	-24.75	-24.75	-24.75	-24.75	-24.75	-24.75	-24.75	-24.75	-24.75	-24.75	-24.75	-24.75	-24.75	-24.75	-24.75	-24.75	-24.75	-24.75	-24.75	-24.75	-14.74	0	6
7		0	0	-24.75	-24.75	-24.75	-24.75	-24.75	-24.75	-24.75	-24.75	-24.75	-24.75	-24.75	-24.75	-24.75	-24.75	-24.75	-24.75	-24.75	-24.75	-24.75	-24.75	-14.74	0	7
8		0	0	-24.75	-24.75	-24.75	-24.75	-24.75	-24.75	-24.75	-24.75	-24.75	-24.75	-24.75	-24.75	-24.75	-24.75	-24.75	-24.75	-24.75	-24.75	-24.75	-24.75	-14.74	0	8
9		0	0	-24.	-24.	-24.	-24.	-24.	-24.	-24.	-24.	-24.	-24.	-24.	-24.	-24.	-24.	-24.	-24.	-24.	-24.	-24.	-24.	-14.	0	9

				75	75	75	75	75	75	75	75	75	75	75	75	75	75	75	75	75	75	75	75	74		
10		0	0	-24.775	-24.775	-24.775	-24.775	-24.775	-24.775	-24.775	-24.775	-24.775	-24.775	-24.775	-24.775	-24.775	-24.775	-24.775	-24.775	-24.775	-24.775	-24.775	-24.775	-14.774	0	10
11		0	0	-24.775	-24.775	-24.775	-24.775	-24.775	-24.775	-24.775	-24.775	-24.775	-24.775	-24.775	-24.775	-24.775	-24.775	-24.775	-24.775	-24.775	-24.775	-24.775	-24.775	-14.774	0	11
12		0	0	-24.775	-24.775	-24.775	-24.775	-24.775	-24.775	-24.775	-24.775	-24.775	-24.775	-24.775	-24.775	-24.775	-24.775	-24.775	-24.775	-24.775	-24.775	-24.775	-24.775	-14.774	0	12
13		0	0	-24.775	-24.775	-24.775	-24.775	-24.775	-24.775	-24.775	-24.775	-24.775	-24.775	-24.775	-24.775	-24.775	-24.775	-24.775	-24.775	-24.775	-24.775	-24.775	-24.775	0	0	13
14		0	0	-24.775	-24.775	-24.775	-24.775	-24.775	-24.775	-24.775	-24.775	-24.775	-24.775	-24.775	-24.775	-24.775	-24.775	-24.775	-24.775	-24.775	-24.775	-24.775	-24.775	0	0	14
15		0	0	-24.775	-24.775	-24.775	-24.775	-24.775	-24.775	-24.775	-24.775	-24.775	-24.775	-24.775	-24.775	-24.775	-24.775	-24.775	-24.775	-24.775	-24.775	-24.775	-24.775	0	0	15
16		0	0	-24.775	-24.775	-24.775	-24.775	-24.775	-24.775	-24.775	-24.775	-24.775	-24.775	-24.775	-24.775	-24.775	-24.775	-24.775	-24.775	-24.775	-24.775	-24.775	-24.775	0	0	16
17		0	0	-24.775	-24.775	-24.775	-24.775	-24.775	-24.775	-24.775	-24.775	-24.775	-24.775	-24.775	-24.775	-24.775	-24.775	-24.775	-24.775	-24.775	-24.775	-24.775	-24.775	0	0	17
18		0	0	-24.	-24.	-24.	-24.	-24.	-24.	-24.	-24.	-24.	-24.	-24.	-24.	-24.	-24.	-24.	-24.	-24.	-24.	-24.	-24.	0	0	18

				75	75	75	75	75	75	75	75	75	75	75	75	75	75	75	75	75	75	75	75			
19		0	0	-24.75	-24.75	-24.75	-24.75	-24.75	-24.75	-24.75	-24.75	-24.75	-24.75	-24.75	-24.75	-24.75	-24.75	-24.75	-24.75	-24.75	-24.75	-24.75	-24.75	0	0	19
20		0	0	-24.75	-24.75	-24.75	-24.75	-24.75	-24.75	-24.75	-24.75	-24.75	-24.75	-24.75	-24.75	-24.75	-24.75	-24.75	-24.75	-24.75	-24.75	-24.75	-24.75	0	0	20
21		0	0	-24.75	-24.75	-24.75	-24.75	-24.75	-24.75	-24.75	-24.75	-24.75	-24.75	-24.75	-24.75	-24.75	-24.75	-24.75	-24.75	-24.75	-24.75	-24.75	-24.75	0	0	21
22		0	0	0	0	0	0	0	0	0	0	0	0	0	0	0	0	0	0	0	0	0	0	0	0	22
23																										23
24																										24
