# Supplementary figures and images for: Novel Insights into MEG3/miR664a-3p/ADH4 Axis and Its Possible Role in Hepatocellular Carcinoma from an in Silico Perspective
Source: Genes (Basel). 2022 Nov 30;13(12):2254. doi: 10.3390/genes13122254 (PMC9778073; doi:10.3390/genes13122254)

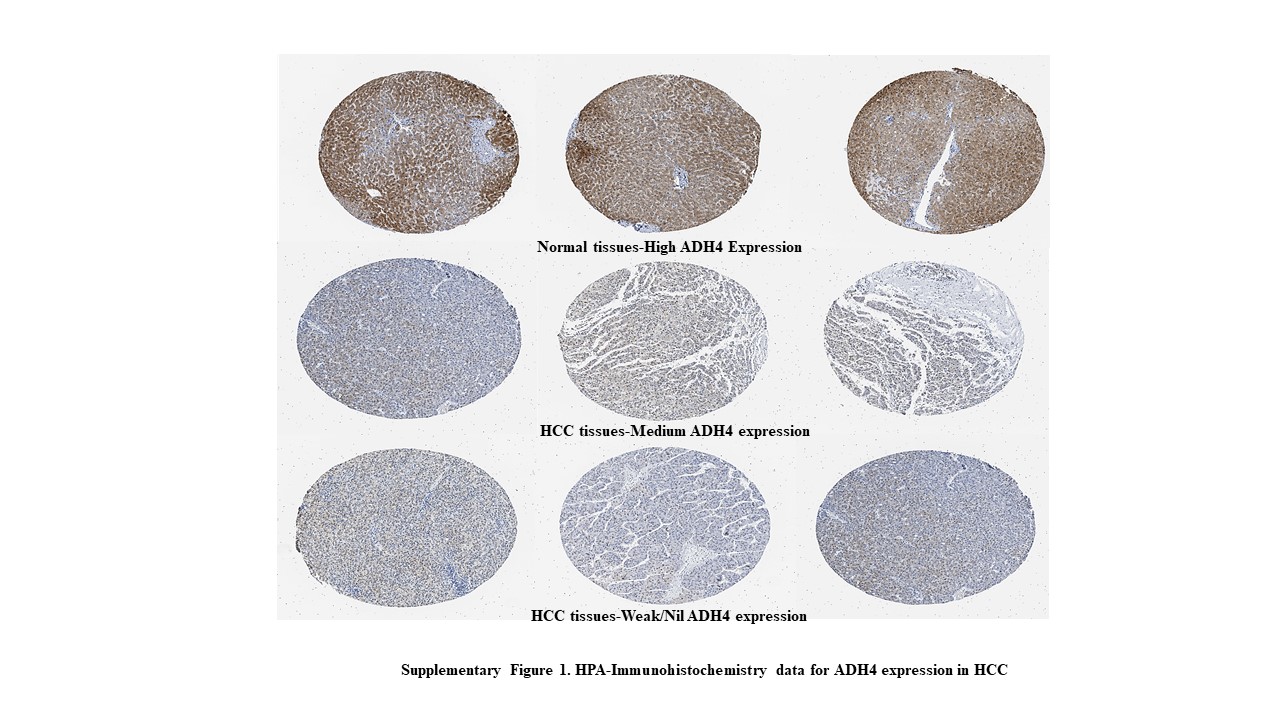

Supplement: Supplementary file 1 [file genes-13-02254-s001.zip › Figure S1.jpg]
